# Analysis of Landscape Characteristics and Influencing Factors of Residential Areas on the Qinghai–Tibet Plateau: A Case Study of Tibet, China

**DOI:** 10.3390/ijerph192214951

**Published:** 2022-11-13

**Authors:** Dingwei Niu, Lucang Wang, Fuwei Qiao, Wei Li

**Affiliations:** 1 College of Geography and Environmental Science, Northwest Normal University, Lanzhou 730070, China; 2 College of Economics, Northwest Normal University, Lanzhou 730070, China

**Keywords:** residential area, landscape characteristics, influencing factors, Tibet

## Abstract

The Qinghai–Tibet Plateau is the largest ecological barrier and one of the most vulnerable areas of the ecological environmental system. However, the increasing frequency of human activities in the Qinghai–Tibet Plateau has led to strong interference. Residential areas are the main places in which human activities are carried out and, as such, can effectively reflect the intensity of activities. Based on this, this research takes the Tibet Autonomous Region as the study area and analyzes the distribution characteristics of Tibetan residential areas using Zipf’s law and various landscape indices, as well as discussing the influences of altitude, hydrology, ecological environment, and location on residential area distribution. The obtained results indicate the following: (1) The residential areas in Tibet basically conform to the rank–size principle. The residential areas in central and northwest Tibet are concentrated in size distribution, and the relatively large residential areas are prominent, while the residential areas in the eastern Hengduan mountain region are relatively balanced in size distribution. (2) The landscape index results demonstrate that the counties with an unbalanced distribution of residential areas are mainly concentrated in the northwest of Tibet, while the residential areas in the counties and regions where the administrative stations of each prefecture-level city (or region) are located tend to present a polarization phenomenon, with large patches. The area distribution of residential areas showed a “medium–high–low” pattern from southeast to northwest. The residential areas in eastern Tibet have a high degree of fragmentation and a low degree of aggregation, while the residential areas in northwest Tibet have a low degree of fragmentation and a relatively high degree of aggregation. (3) The residential areas in Tibet are most concentrated in the altitude range of 3000–5000 m above sea level and their water affinity and road–affinity are strong, with the distribution of residential areas within 500 m of roads and water networks accounting for more than one-quarter. The vegetation coverage in the residential areas is low, inconsistent with the surface vegetation coverage rate over the whole of Tibet.

## 1. Introduction

The Qinghai–Tibet Plateau area is the largest ecological barrier and one of the most vulnerable areas of the ecological environmental system, known as “the Roof of the World”, “the Water tower of Asia”, and “the third pole of the Earth” [1]. The Qinghai–Tibet Plateau has long been a focal point for scientific research and national strategies. To date, relevant research on the Qinghai–Tibet Plateau has mainly focused on its natural environment and geological structure, such as climate change [2,3,4], plateau vegetation [5,6,7], plateau strata and tectonic movements [8,9,10], ecological risks [11,12], and so on. With the increasing urbanization of the Tibetan plateau, studies related to human activities, such as the settlement pattern of the plateau population [13], the development of plateau cities [14,15], and residential environmental evaluation [16] have gradually emerged. Landscape change often generates a variety of environmental problems and exacerbates regional landscape competition and conflict, while settlement landscape features provide a good record of the spatial patterns of earth surface features that have been altered by human activities [17]. 

As the hinterland of the Qinghai–Tibet Plateau, Tibet is the highest and largest plateau in the world. Despite the low-pressure hypoxia; low average temperature, relative humidity and rainfall; and high wind speed and solar radiation in Tibet, the local population has developed lifestyles and measures to adapt to the harsh thermal environment through long-term mutual adaptation. In recent years, the long-term evolutionary relationship between human activities and natural environmental interactions on the Tibetan Plateau has been widely discussed, and Tibet has become the focus of extensive discussion. For example, Zhu et al. [18] have quantitatively analyzed the effects of human activities and climate change on vegetation coverage. Gao et al. [19] studied the response of alpine grassland conditions in the northern Qinghai–Tibet Plateau to human activities and found that the impact of roads on alpine grasslands was stronger than that of residential areas. Some scholars have also conducted relevant studies on the social economy and residential areas in Tibet, mainly focusing on the form and distribution of residential areas [20], optimization of spatial layout [21], ecological adaptations [22,23], and influencing factors [20]. For example, Qiao et al. [20] studied the spatial agglomeration characteristics of residential areas in Tibet and pointed out that population distribution and GDP (Gross Domestic Product) were important factors affecting the distribution of residential areas. Chen et al. [24] simulated the thermal environment of residential areas with different landscape elements in Lhasa by the orthogonal experimental design method. Wang et al. [22] analyzed four ecological construction modes of residential buildings in the Kangba area of Tibet, and they pointed out that the layout of Tibetan villages can be divided into centripetal and decentralized layouts. Li et al. [25] developed a scale to measure the vulnerability of the Tibetan population to high temperatures and explored the impact of high temperatures on the population of the region. Qin et al. [26] analyzed the residential model under the influence of folk culture and religious culture, based on an investigation of the natural environment, life, and production modes. Gao et al. [27] comprehensively evaluated the quality of rural human settlements, and they discussed the evolution and influencing factors of rural human settlements in the Qinghai–Tibet Plateau. Jin et al. [28] used the nearest neighbor index, kernel density estimation, and spatial autocorrelation to reveal the spatial distribution characteristics of traditional villages in Tibet, China, and found that traditional villages present the spatial distribution characteristics of ‘large dispersion and small collection’. With the continuous deepening of human activities such as regional urbanization and industrialization in the Tibetan Plateau, the regional surface land structure and ecosystem have changed [29]. Zhao et al. [30] explored the influencing factors affecting the distribution of Yarlung Tsangpo River source settlements on the Qinghai–Tibet Plateau.

Therefore, some scholars have begun to evaluate the suitability of the living environment and ecosystem health in this area. For example, using a quantitative evaluation model of construction land and cultivated land suitability based on raster data, Xu et al. [31] proposed a universal algorithm for key parameters such as air density, temperature, slope suitability, and soil erosion resistance of slope farmland, and quantitatively analyzed the suitability grade and spatial distribution of Milin County on the Qinghai–Tibet Plateau. Shi et al. [32] comprehensively assessed the health status of the ecosystem in the Qinghai–Tibet Plateau animal husbandry area based on a combination of multiple models. Chen et al. [33] assessed the potential risk of the rapid expansion of inland lakes on the Tibetan Plateau to the human habitat. With the contradiction of the man-land relationship in Tibet becoming more and more prominent, urbanization and sustainable development of urban or rural settlements have become hot spots. For example, Dong et al. [34] assessed the urban ecological sustainability of Tibetan prefecture-level cities based on an improved ecological footprint model. Wang et al. [35] explored the impact of urban agglomeration expansion on landscape connectivity on the Tibetan Plateau and found that urbanization leads to changes in landscape morphology and connectivity. Li et al. [36] studied the spatial and temporal evolution characteristics of rural settlements on the Tibetan plateau and provided policy recommendations for rational planning of rural settlements.

Existing research has mainly focused on the ecological barrier of the Qinghai–Tibet Plateau, as the “third pole of the Earth”. As an important area for Chinese characteristic culture protection, few people have paid attention to the landscape distribution characteristics of residential areas; however, it is very clear that landscape planning can contribute to both landscape aesthetics and ecosystem services [37]. Moreover, the Qinghai–Tibet Plateau is an area with a vulnerable ecological environment. With the acceleration of the urbanization process in Tibet, the interference of human activities on the ecological environment is becoming more and more intense. Based on the above, we selected Tibet, the hinterland of the Qinghai–Tibet Plateau, as the study area. We used Zipf’s law and landscape indices to explore the landscape characteristics of Tibetan residential areas and determine the main cluster areas of Tibetan people in order to provide an important basis for the formulation of ecological protection policies. This study intends to accomplish the following three objectives: (1) to determine the order–scale characteristics of Tibetan residential areas; (2) to present the landscape characteristics of Tibetan residential areas; (3) to reveal the influence of natural elements (e.g., altitude, ecological environment, hydrological conditions) and human factors on the spatial distribution of residential areas.

## 2. Materials and Methods

### 2.1. Research Framework

**Figure 1:**
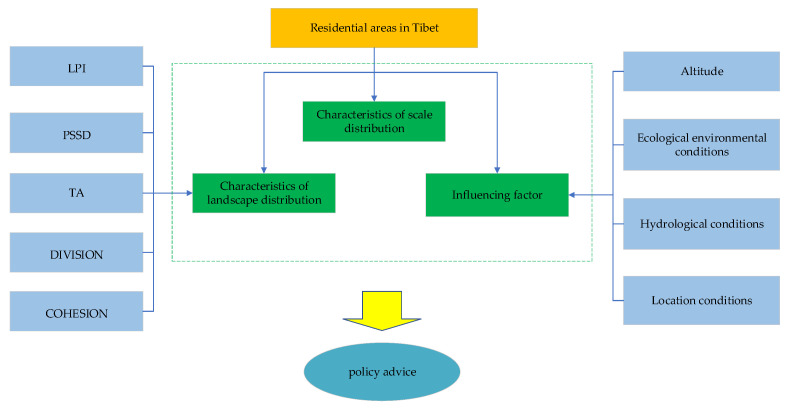
Research framework.

The research framework of the main part of this paper mainly includes three aspects (Figure 1): First, the rank–size rule is used to reveal the scale grade characteristics of Tibetan residential areas. Second, landscape indices (mainly including the total area of patch, TA); standard deviation of patch size, PSSD; cohesion index of patch, COHESION; patch density, PD; Largest patch index, LPI; and degree of landscape division, DIVISION) were used to describe the landscape characteristics of residential areas. Then, the effects of altitude, eco-environmental conditions, hydrological conditions, and location conditions on the spatial distribution of residential areas are discussed. The specific methods were as follows: (1) The altitude of Tibet was divided into 11 grades with 500 m as equal spacing, and the respective area distribution of residential areas at different altitudes was determined, in order to reveal their correlation. (2) Vegetation indices, such as the NDVI (Normalized Difference Vegetation Index) provide a simple, effective, and empirical means to measure the condition of vegetation. Therefore, utilize the NDVI as an index to measure the living environment characteristics of residential areas. This paper divides NDVI into ten grades and calculates the area distribution of residential areas under different grades. (3) The influence of water and traffic conditions on the distribution of residential areas under different distance conditions were statistically analyzed at 500 m intervals. Finally, according to the analysis of different characteristics of Tibetan residential areas and the actual situation in Tibet, this paper puts forward relevant policy recommendations.

### 2.2. Study Area

**Figure 2:**
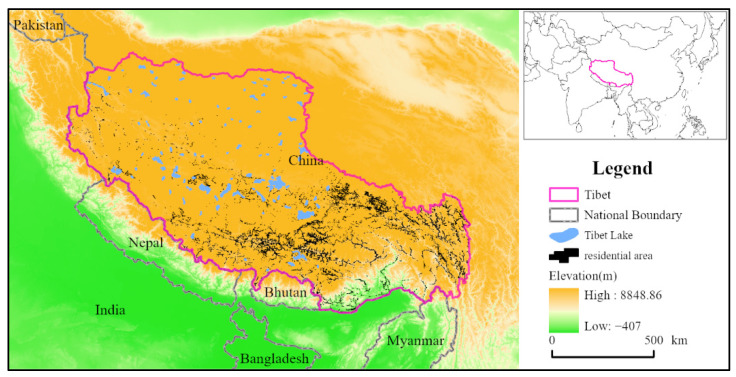
Overview of the study area in the background of Tibet.

The Tibet Autonomous Region covers an area of 1.2 million square kilometers, accounting for one-eighth of China’s total area (Figure 2). Tibet is the main part of the Qinghai–Tibet Plateau, with an average altitude of more than 4500 m. Its range refers to the area between the south of the Kunlun Mountains and the north of the Himalayan Mountains. Its west part extends to the Karakoram Mountains and its east part to the Hengduan Mountains. The northern part of Tibet is a plateau, bounded by the Kunlun Mountains, the Gangdise Mountains, and the Nianqing Tanggula Mountains. The southern part of Tibet contains the Yarlung Zangbo River and other tributary valleys of Tibet, while the southeast is a series of high mountain gorges running north-south. There are now 6 prefecture-level cities, 1 prefecture, and 74 counties (also including districts) in the Tibet Autonomous Region. According to the seventh national census, at the end of 2020, the total permanent resident population of the Tibet Autonomous Region was 3.64 million, with an average population density of 2.98 ind·km^−2^. The Tibetan population in the region is about 3.1379 million, accounting for more than 86%. Regionally, there are 867,900 people in Lhasa, 798,200 in Xigaze City, 761,000 in Qamdo City, 238,900 in Nyingchi City, 354,000 in Shannan City, 504,800 in Naqu City, and 123,300 in Ngari Prefecture [38]. The GDP of the region exceeded 200 billion yuan in 2020. In terms of land-use, the types of land-use in Tibet mainly include grassland, forest land, and unused land, with these three types of land accounting for more than 90% of the total land area in the region. However, the growth rate of urban and rural construction land has been accelerating over the past 20 years [39].

(1) Characteristics of typical residential areas

**Figure 3:**
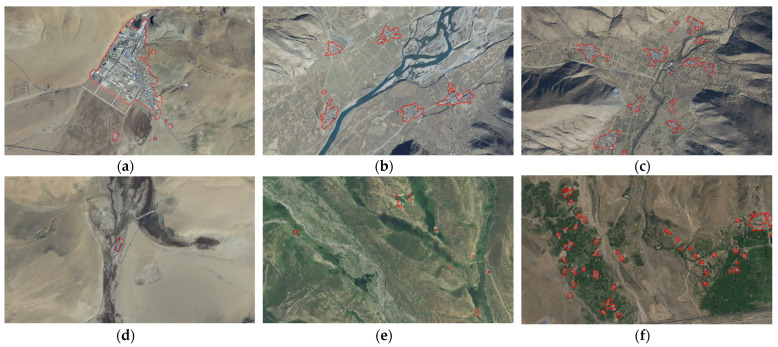
Satellite maps of typical residential areas: (**a**) Large-patch dominant type; (**b**) Low-density and cluster type; (**c**) Medium-density and cluster type; (**d**) Small-patch isolated type; (**e**) Low-density and scattered-point type; (**f**) High-density and scattered-point type.

Based on the research of Qiao et al. [40], this paper divides the landscape characteristics of Tibetan residential areas into six types (Figure 3): large-patch dominant type, low-density and clustered type, medium-density and clustered type, small-patch isolated type, low-density and scattered-point type, and high-density and scattered-point type. Specifically, the large-patch dominant type is characterized by the existence of single-core giant patches in the region, with a scale far larger than that of surrounding residential areas; most of such areas are distributed in government offices in cities and counties. The low-density and clustered type indicates that the residential area is clustered and the distribution density is very low. In the medium-density and clustered type, the residential areas are mostly irregular and densely distributed. The feature of the small-patch isolated type is that there is only a small individual residential area within a relatively large area. The characteristics of the low-density and point-scattered type is that the distances between residential areas in the region are large, while the number of residential areas in the region is small and scattered. The characteristics of the high-density and point-scattered type is that residential areas are not far apart, but are not contiguous, with scattered shapes and a high degree of fragmentation.

(2) Topographic characteristics

Tibet, with an average altitude of more than 4000 m, is the main part of the Qinghai–Tibet Plateau. The terrain here is complex and can be divided into four different natural areas: The northern Tibetan Plateau, which is located between the Kunlun Mountains, Tanggula Mountains, Gangdise Mountains, and Nianqing Tanggula Mountains, accounting for two-thirds of the total area of the autonomous region; the south Tibet Valley, located between the Gangdise Mountains and the Himalayas, i.e., the Yarlung Zangbo River and its tributaries run through this area; the alpine gorge area in eastern Tibet, comprised of a series of alpine deep valleys that gradually change from east-west to north-south, and which is part of the famous Hengduan Mountains; and the Himalayas on the south side, composed of many parallel mountains with an approximate east-west direction. Its main part is on the boundary between China, India, and Nepal, with an average altitude of more than 6000 m. The world’s highest peak, Everest, is located here. Within more than 5000 square kilometers around it, there are 4 peaks above 8000 m and 38 peaks above 7000 m.

(3) Traffic conditions

**Figure 4:**
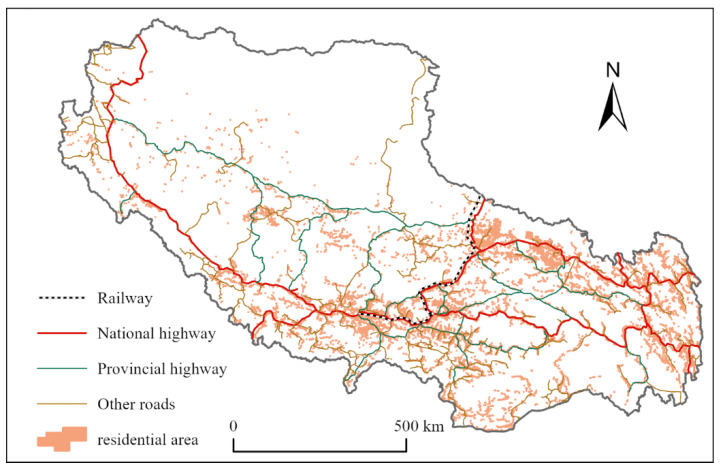
Spatial location map of Tibetan residential area and road network.

As an important part of the Qinghai–Tibet Plateau, before the 1950s, due to the influence of natural conditions, transportation in Tibet was extremely backward and closed [41]. There was no road in the area, and transport was carried out by people on their backs and livestock. Roads in Tibet were developed after the 1950s. The construction of the Qinghai–Tibet Highway and the Sichuan–Tibet Highway ended the history of Tibet having no official roads. Later, the Xinjiang–Tibet, Yunnan–Tibet, and China–Nepal highways were built, as well as regional trunk roads and numerous county, township, and frontier roads. At present, Tibet has a highway network consisting of national roads as the main trunk and provincial roads as the support, followed by county, township, special, and frontier roads. The main highways in Tibet include the Qinghai–Tibet Highway, Xinjiang–Tibet Highway, Sichuan–Tibet Highway, Yunnan–Tibet Highway, and China–Nepal Highway, while the railways include the Qinghai–Tibet Railway and Lhasa–Xigaze Railway. Figure 4 shows the location relationship between the residential areas in Tibet and the transportation network.

(4) Hydrological conditions

**Figure 5:**
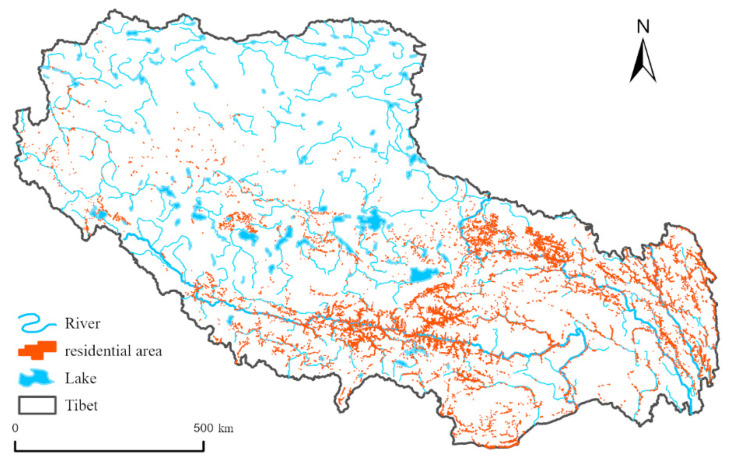
Spatial location map of Tibetan residential areas and waters.

In Tibet, there are more than 20 rivers with a basin area of more than 10,000 square kilometers, and more than 100 rivers with a basin area of more than 2000 square kilometers. The famous rivers include the Jinsha River, Nujiang River, Lancang River, and Yarlung Zangbo River. The Ganges River, the Indus River, the Brahmaputra River, the Mekong River, the Salween River, the Irrawaddy River, and other rivers have the upper source here. The Yarlung Zangbo River is the largest river in Tibet, which originates from the northern foot of the Himalayas. It is 2057 km long in China and covers an area of more than 240,000 km^2^. Tibet has the largest area of lakes in China, with a total area of 23,800 km^2^, accounting for about 30% of the total lake area in China. Tibet has more than 1500 lakes, including fewer freshwater lakes, saltwater lakes, and salt lakes in the majority of which 47 have an area higher than 100 square kilometers. Figure 5 shows the spatial location of Tibetan residential areas and waters.

(5) Ecological environmental conditions

**Figure 6:**
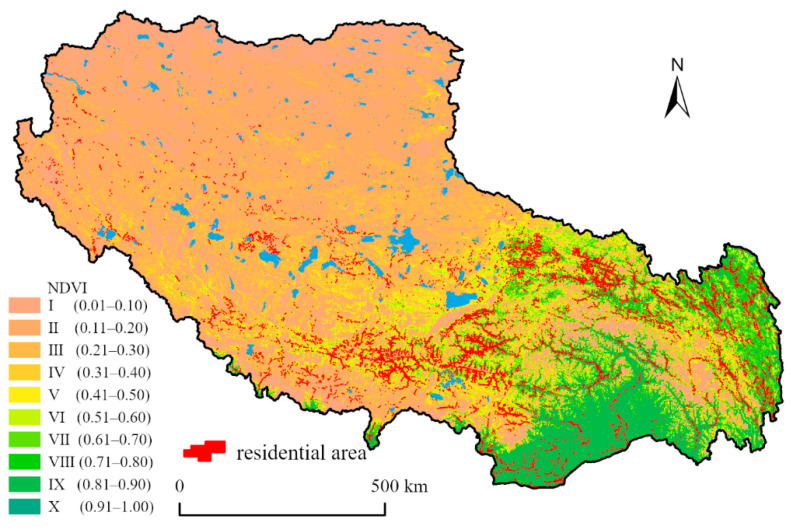
Spatial location map of Tibetan residential area and NDVI.

Affected by natural conditions, most areas of Tibet’s ecological environment belong to ecologically fragile areas, especially in the northern Tibetan plateau, with large bare land distribution and low vegetation coverage. Comparatively speaking, the valley plain and Hengduan mountain areas in the southeast, with lower altitudes, have better ecological environment conditions and high vegetation coverage (Figure 6). Therefore, Tibet’s ecological environment conditions are gradually getting better from northwest to southeast [42].

(6) Other conditions.

Residential areas are the result of the interaction between the natural environment and human activity under a specific geographical environment and social economic background [43]. The Tibetans in Tibet account for over 86% of the total population, who share Buddhism beliefs. As such, most of them live close to temples, and the existence of a Sacred Mountain has a certain effect on the distribution of Tibetan residential areas. In addition, existing studies have shown that religion is an important driving force for economic growth and promotes regional economic growth, which also increases the complexity of studying the social activities of residents in this region [44,45].

### 2.3. Data Sources

The research area of this paper was the Tibet Autonomous Region, and the analysis unit was mainly the county-level administrative region. The administrative boundary of the research area was based on the administrative division of the Tibet Autonomous Region in 2017, including 6 prefecture-level cities, 1 prefecture, and 74 counties (also including districts). The standard map used was from the brief administrative map of the Tibet Autonomous Region and the Traffic Edition of Tibet Autonomous Region, both published by the Bureau of Surveying and Mapping of Tibet Autonomous Region [46]. Residential areas were obtained through visual interpretation of the LocaSpaceViewer software (Zhongke Tuxin (Suzhou) Technology Co., Ltd., Suzhou, China). The DEM (Digital Elevation Model) data for Tibet, with 30 m resolution, were originally obtained from the geospatial data Cloud website of the Computer Network Information Center, Chinese Academy of Sciences, and later pruned for the research [47]. It should be noted that as the residential areas in Chengguan District and Duilongdeqing District of Lhasa are concentrated and developed as a whole, in order not to destroy the integrity of the patches, Chengguan District and Duilongdeqing were combined into one area (called the Lhasa urban area) for the relevant analyses.

### 2.4. Methods

#### 2.4.1. Rank–Size Principle

The rank–size rule was proposed by Frank Auerbach in 1913, which refers to the relationship between the size of a city and the rank of the city in the order of population size in all cities of the country. It can help to examine the size distribution of a city system according to the relationship between city size and city rank order. The rank–size principle can describe the city size distribution rule well, and it has been proved that it can also be applied to assess an urban land-use scale. F. Auerbach pointed out that the city size distribution can be described using the Pareto distribution. In 1949, Zipf developed and constructed the theoretical basis, forming Zipf’s Law. The generalized expression of Zipf’s Law is the urban rank–size rule, with the formula as follows: [48]:

(1) $$S_{i}=S_{0}\cdot R_{i}^{-q}$$

For sake of clarity of the result, the natural logarithm transformation is usually conducted on the Catro formula, as follows:

(2) $$\mathrm{Ln}S_{i}=\mathrm{Ln}S_{0}-q\mathrm{Ln}R_{i}$$

where *R_i_* is the rank order of city *i,* sorted by size from largest to smallest; *S_i_* is the city size in the rank order *R_i_*; *S*_0_ is the theoretical value of the first city size, and the parameter *q* is often referred to as the Zipf index.

The value of *q* can reflect the equilibrium degree of the city system. When *q* > 1, it indicates that the city size distribution in the region is relatively concentrated, with large size cities having high rank being prominent, while the small and medium-sized cities have relatively low rank, being less developed. Such cities vary greatly in size. When *q* < 1, it indicates that the city size distribution in the region is relatively scattered, the scale of the high-rank cities is not very prominent, and the small and medium-sized cities are relatively developed. There is little difference in the distribution of city sizes. When *q* is close to 1, it means that the ratio between the scale of the largest city and the scale of the smallest city in the region is close to the total number of cities in the region; thus, the city size distribution is close to Zipf’s ideal state, and the proportion of cities of each size level is reasonable.

#### 2.4.2. Landscape Index

The residential landscape is a mosaic of natural and cultural patches of different sizes, shapes, and combinations. Landscape indices come from landscape ecology, which can highly condense landscape pattern information and reflect certain characteristics in terms of its structural composition and spatial configuration. In this paper, the total area of patch (TA), standard deviation of patch size (PSSD), cohesion index of patch (COHESION), patch density (PD), largest patch index (LPI), and degree of landscape division (DIVISION) are selected to study the scale and spatial distribution characteristics of a residential area. The specific formulas are provided in Table 1.

## 3. Results

### 3.1. Spatial Distribution Based on Rank–Size Principle

By implementing logarithmic linear regression between the rank of residential patches and the residential patch area in Tibet, it was determined that the fitting coefficient R^2^ of the seven prefecture-level cities and seventy-four counties in Tibet was above 0.85, indicating a good fit level. The results indicated that the distribution of the scale of a residential area at the prefecture-level and county-level in Tibet basically conforms to the rank–size principle. In order to have strong comparability, regions with the Zipf index fitting R^2^ reached more than 95% were compared in this paper (Table 2). It should be noted that, due to the different geographical conditions, economic development levels, population, resources, and environmental conditions in different regions, the distribution of the scale of residential patches in Tibet also reflects such differences.

From the perspective of prefecture-level cities, the Zipf index of Ngari Prefecture was the largest (1.4947) among the seven prefecture-level cities in Tibet, while that of Lhasa was the second-largest (1.0006). The lowest Zipf index was 0.6555 in Chamdo City. These results show that the scale distribution of residential patches in the Ngari Prefecture was concentrated, with residential patches being prominent, while the middle and small residential patches were not developed enough. The relative difference in the residential patch was large. This is because the Ngari Prefecture is a place where the Himalayan mountains, Gangdise Mountains, and other mountains meet, marking it as one of the regions with the lowest population densities in the world, with a small distribution of residential areas, mainly concentrated in the places where a county or administrative seats are stationed. Therefore, the Zipf index of this area obtained was large, but the total scale of residential areas was not the largest. The situation in Lhasa was different. As the capital of the Tibet Autonomous Region, Lhasa is the political, economic, cultural, scientific, and educational center of Tibet. Due to the strong siphon effect, the urban scale of Lhasa is well-developed, which is much larger than the development of other towns and counties, so, the Zipf index was relatively large. The residential areas in Chamdo are mainly distributed along the valley bottom of the Nu River, Lantsang River, and Jinsha River, and the scale equilibrium of the residential patch is good, making the Zipf index small.

**Figure 7:**
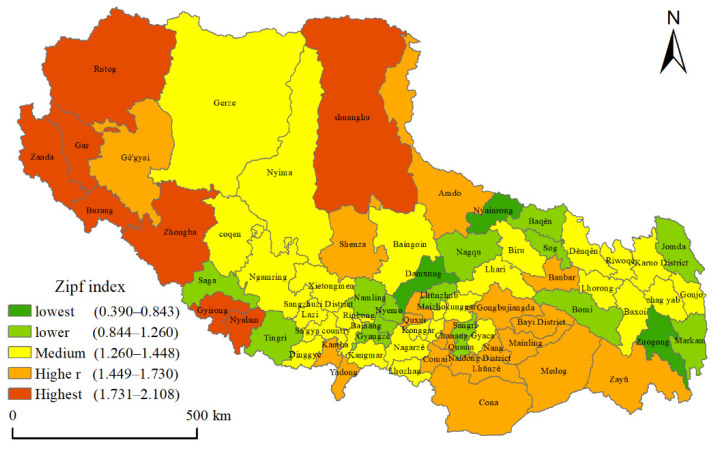
Zipf index spatial distribution map.

The Zipf index values of counties were divided into five levels using the Natural Breaks method (Figure 7). In general, the counties in Ngari Prefecture were the main distribution areas with high Zipf values. There were eight areas with a high Zipf index, and four of them were concentrated in the Ngari Prefecture. Shannan City and Nyingchi City were also the distribution areas with relatively high Zipf index, accounting for 57% of the total high values. The areas with a high value are mountainous interlacing locations, with sparse population distribution and concentrated residential areas, while being relatively stunted in small and medium-sized residential areas. The low Zipf value areas were distributed in Lhasa, northeast, and southwest of Xigaze, east of Nagqu, and southeast of Chamdo. The residential area distribution was greatly affected by rivers; however, the narrow river valleys are not conducive to the formation of large residential areas.

### 3.2. Spatial Distribution Based on Landscape Indices

#### 3.2.1. Spatial Distribution of Residential Patch Area and Density

The changes in the residential patch scale include the changes in the total land-use scale and the changes in the residential patches themselves. The TA, MPS, and LPI indices were used to characterize the scale characteristics of the residential patches. Table 3 shows the corresponding landscape index calculation results for residential areas in Tibet. The total area (TA) of the whole of Tibet was 795.96 km^2^, and the TA of Lhasa City was 195.80 km^2^, accounting for 24.61%. Shannan City’s TA was 167.07 km^2^, while that of Chamdo City was 102.78 km^2^. The total residential area in Ngari Prefecture was the lowest, at 29.98 km^2^, accounting for only 3.77%. The PSSD index can reveal the uniformity of the residential patch area distribution. The larger the PSSD value, the stronger the polarization in the distribution of regional residential areas. The PSSD results of Lhasa showed that the area difference of residential areas in Lhasa was the most tremendous and so, the area of residential patches was polarized. The LPI value for Lhasa was also the largest, indicating that the two-level differentiation of residential areas in Lhasa was caused by the prominent position of large patch areas. The PSSD and LPI in Ngari Prefecture were second only to those of Lhasa City, and the LPI value was higher than the mean value for the whole of Tibet, indicating that the polarization distribution phenomenon also existed in the Ngari region, due to the large residential patch. The PSSD and LPI values for Chamdo City were the smallest, indicating that the distribution of residential areas in Chamdo City was in a balanced situation. The PSSD values for Shannan City, Xigaze City, Nyingchi City, and Nagqu City, were all lower than the mean value for the whole region of Tibet, and, of these cities, the LPI value for Nagqu City was the highest.

**Figure 8:**
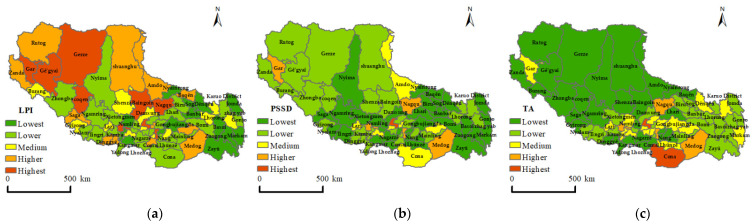
LPI, PSSD, and TA index’s spatial distribution maps by county. (**a**) LPI; (**b**) PSSD; and (**c**) TA LPI. LPI is largest patch index; PSSD is standard deviation of patch size; TA is the total area of patch.

The TA, PSSD, and LPI indices of the 74 counties in Tibet were divided into five levels using the Natural Breaks method (Figure 8). The TA results showed that the residential areas were mainly distributed in the eastern part of Tibet. Cona County in Shannan City and the southern part of Medog County in Nyingchi City have low altitudes, with low terrain, thus maintaining a humid sub-tropical mountainous sub-humid climate with good climate conditions. Therefore, they presented a greater residential area distribution. The residential area of Cona County was higher than that of Nedong District (in Shannan City, China), while that of Medog County was higher than that of Bayi District (in Nyingchi City, China). The residential areas of the rest of the five counties (or districts) where prefecture governments are located were higher than those of counties in other corresponding prefecture-level cities. Ngari Prefecture, Nagqu City, and most counties of Xigaze City presented low-TA value areas. The PSSD results demonstrated that the polarization phenomenon of the urban residential area in Lhasa was the most obvious, followed by Gar County, Samzhubze District, Seni District, Nedong District, and Medog County. Among them, Gar County, Samzhubze District, Seni District, and Nedong District were the administrative stations of corresponding prefectural cities (or regions). The balanced distribution characteristics of all counties’ residential areas in Chamdo were obvious, mainly because they are located in the Hengduan Mountain area, with an alternative distribution of mountains and rivers, and highly undulating terrain. Therefore, in this region, it is difficult to form large residential areas. The LPI results indicated that the counties with an unbalanced distribution of residential areas were mainly concentrated in the northwest of Tibet, and the residential areas in the counties and districts where the prefecture-level cities’ administrative stations are located presented higher polarization phenomena with large patches.

#### 3.2.2. Distribution of DIVISION and COHESION

The DIVISION (degree of landscape division) and COHESION (cohesion index of patch) indices were used to measure the fragmentation degree and aggregation characteristics of the Tibetan residential area patch distribution, respectively. Table 4 shows that the DIVISION index for the whole of Tibet was 0.9849, while the DIVISION value of Chamdo City was the largest, indicating that the distribution of residential area in Chamdo was the most fragmented. The DIVISION index of residential areas in Xigaze City was basically consistent with the value for the whole of Tibet. The DIVISION values for Lhasa and Ngari Prefecture were 0.7815 and 0.8813, respectively, significantly lower than those of other cities. The COHESION results demonstrated that the COHESION values for the whole of Tibet and all the prefecture-level cities in Tibet were above 99%, indicating that the distribution of residential areas in-all regions presented cohesion characteristics. The values for Lhasa and Ngari Prefecture were 99.75% and 99.63%, respectively, slightly higher than those of other cities, indicating that the cohesion characteristics of Lhasa and Ngari Prefecture were more obvious. Meanwhile, the COHESION value for Chamdo City was the smallest (99.21%), indicating that the cohesion degree of Chamdo was relatively the smallest. The reason for this may be that most of Chamdo City is located in the longitudinal ridge and valley belt, mainly in the type of plateau landform, with a high altitude. The ground fails to form a wide valley due to the large undulation of plateau cutting, which is not conducive to the formation of large residential areas. Shannan City is a typical southern Tibet valley city. In the north of Shannan City, the residential areas are mainly distributed in the mountains and valleys, without forming large-scale contiguous residential areas. In the south of Shannan City, there are plains at the southern foot of the Himalayas, but there are no large urban residential areas. Therefore, the division degree of residential areas in Shannan City is relatively large. Mainly due to the strong siphon effect, the distribution of residential areas in Lhasa City is concentrated and contiguous, while the small number of people in Ngari Prefecture are all concentrated in the environment which is conducive to survival, due to the role of natural conditions; hence, the degree of division is low in this region.

**Figure 9:**
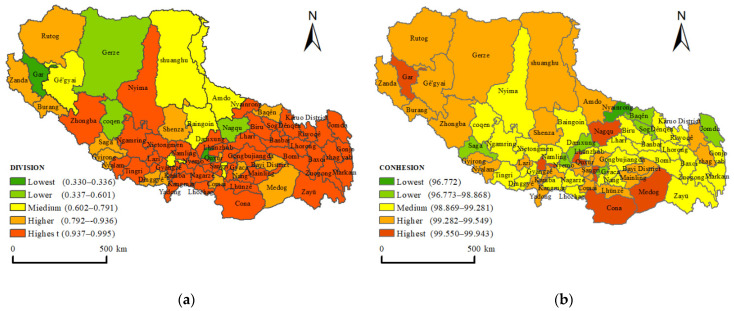
DIVISION and COHESION index spatial distribution maps by county. (**a**) DIVISION is degree of landscape division and (**b**) COHESION is cohesion index of patch.

In order to further explore the division and aggregation degree of residential areas in each county, DIVISION and COHESION in the 74 counties of Tibet were divided into five levels, using the Natural Breaks method, and visualized (Figure 9). The DIVISION results showed that the large fragmentation degree of residential areas was mainly distributed in counties in eastern Tibet. In the whole of eastern Tibet, except for Medog County, Bayi District, Gyaca County, Comai County, Nedong District, Dagze District, and the Lhasa urban area, the fragmentation degree of residential area in other counties was large, while the fragmentation degree in northern Tibet was low. The degree of division was the lowest in Lhasa City and Gar County, followed by Gerze County, Coqen County, and Seni District. On the whole, the Nagqu area appeared as a concentrated distribution area with a medium degree of division. The COHESION results indicated that the districts (or counties) where their prefecture-level administrative stations are located showed high aggregation characteristics. Ngari Prefecture and the western Nagqu area were the distribution areas with high aggregation degree of residential areas, while the residential area in eastern Nagqu City showed low aggregation characteristics. In general, the counties with a moderate or higher degree of residential area had absolute superiority in number.

### 3.3. Analysis of Factors Influencing Residential Area in Tibet 

#### 3.3.1. Effects of Altitude on Residential Area in Tibet

**Figure 10:**
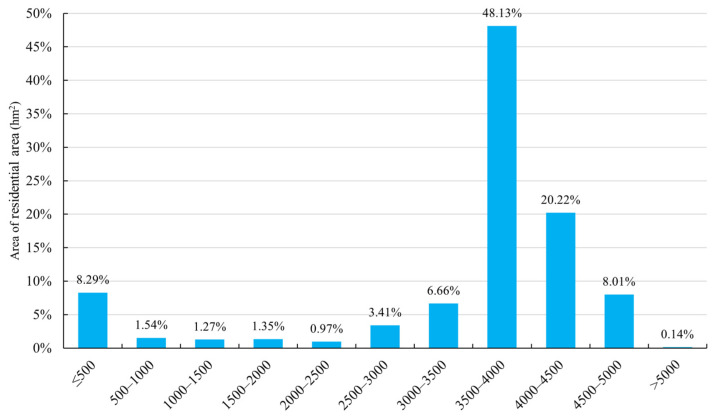
Area statistics of residential areas at different altitudes.

Among the various natural factors, altitude has an important influence on the distribution of residential area. The distribution in number and area of residential area in Tibet presented diversity at different altitudes (Figure 10). From 3500–4000 m in altitude, the number and area distribution of residential areas were the largest, with 33,028 residential areas, accounting for a total area of 42.554 ha, thus having an area proportion of 48.13%. The number and area of residential areas distributed in the altitude zone of 4500–5000 m were second, followed by that from 4000–4500 m. The number of residential areas in these places was 16,105 and 7540.318 ha (accounting for 20.22%), respectively. The total area below 2000 m altitude was about 12%, and there was no residential area distribution above 5500 m altitude. Therefore, the distribution of residential area in Tibet is mainly concentrated in the high-altitude zone from 3500~5000 m, accounting for 89.49% of the residential areas. The number of residential areas in each altitude zone decreased significantly around the peak values of 3500–5000 m. The area above 5500 m altitude basically had no settlement distribution. This is because the average altitude of Tibet is over 4000 m, while those 473 of Nagqu, Ngari, and Xigaze are 4500 m, 4300 m, and 3800 m, respectively. The average altitude of Lhasa is 3700 m, while that of Shannan is 3700 m, Chamdo 3200 m, and Nyingchi 3000 m with the lowest altitude being below 1000 m. The landscape index TA (Table 3) of residential areas indicated that the distribution areas of residential areas are mainly concentrated in Lhasa, Xigaze, and Shannan, where the average altitude exceeds 3500 m. In Chamdo City and Nyingchi City, with an average altitude below 3500 m, the residential distribution accounts for a relatively small proportion. In a word, the distribution of residential areas in Tibet does not show the principle of gradually decreasing when the altitude rises in the whole region. This is mainly because Tibet is located in the core of the Qinghai–Tibet Plateau, with altitudes ranging from less than 1000 m to more than 8000 m. However, the areas at low altitudes (below 1000 m) account for only 1.34%, while the area at high altitudes (3500–5000 m) and extremely high altitudes (above 5000 m) account for 81%. The inequality of areas at different altitudes, therefore, broke the overall principle of the residential area distribution gradually decreasing as the altitude rises, thus presenting a right–biased normal distribution curve.

#### 3.3.2. Ecological Environmental Conditions

At the regional scale, the average NDVI value in Tibet was 0.209, and the vegetation coverage over the whole region was low; however, the difference between regions was great, with a max-min difference of 0.962. The overall vegetation coverage showed a decreasing trend from southeast to northwest. In terms of NDVI in the distribution area of residential areas, the distribution of NDVI values also very greatly differed, ranging from 0 to 0.984. The NDVI was divided into ten grades, as detailed in Table 5. The NDVI values of residential areas were mainly concentrated in grades Ⅱ, Ⅲ, Ⅳ, and Ⅵ. The total area of these four grades was 633.38 ha, accounting for 79.57%. The total proportion of residential area distribution in the Ⅸ and Ⅹ grades, with the best vegetation coverage, was only 1.00%. In short, the distribution of residential area in Tibet is not consistent with that of high-vegetation cover areas. The vegetation in most areas of Tibet is sparse, especially in Ngari Prefecture, most of Nagqu City, and most of Xigaze City. The vegetation indices of these areas were all <0.2, basically making them desert and rock. The vegetation indices of eastern Nagqu City, Lhasa City, Chamdo City, and northern Shannan City were between 0.2 and 0.4. On the eastern edge of Chamdo and the southern edge of Xigaze, the vegetation index reaches above 0.4. The areas with the best vegetation coverage were Nyingchi and southern Shannan, with vegetation indices above 0.6. The main resident distribution areas were Lhasa City, Shannan City, and Xigaze City. Although the vegetation coverage is high in the southern area of Nyingchi City and Shannan City, the total distribution of residential areas accounts for a small proportion of these areas. All of these factors may have caused the distribution of residential areas to be inconsistent with that of high-vegetation cover areas.

#### 3.3.3. Hydrological Conditions

**Figure 11:**
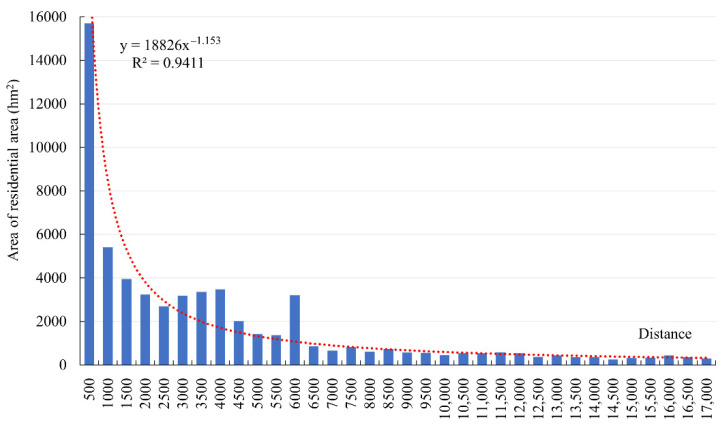
Figure of the total area of residential patch changing with the distance from waters.

This paper analyzed the buffer zone of Tibetan waters (including rivers and lakes) in 500 m intervals. Figure 11 shows that, with a continuous increase in the distance from the river, the area distribution of the residential area in Tibet showed a significant decreasing trend. The total area of residential patches within 500 m was 193.01 ha, accounting for 24.24% of the total area, while the area of the residential areas within 3000 m was 392.70 ha, accounting for 49.33% of the total patch area. These residential areas were mainly distributed in the Yarlung Zangbo River Valley, Nu River Valley, Jinsha River Valley, and Lancang River Valley. In the valley, the altitude is low, with good heat conditions, flat terrain, fertile soil, and sufficient water. The Lhasa River Valley, as one of the five main streams of the Yarlung Zangbo River, is an area where the residential area has accumulated. It provides a suitable living environment for people and is conducive to the development of the economy. The population and city are relatively dense. The areas more than 100 km away from the river, making up 52.93 ha, accounted for 6.5% of the total patch area. These areas were mainly distributed in the northern part of Ngari Prefecture and located in the Southern Qiangtang High lake Basin region, all of which are high mountain valley zones without plains. The mountains are gentle and the terrain slopes from northwest to southeast. There is a lot of glacial meltwater, but it does not form a major river. Thus, the residential areas in Tibet show strong “water affinity” characteristics, especially in the Yarlung Zangbo Valley, Nu River Valley, Jinsha River Valley, and Lancang River Valley. Due to the good terrain, climate, water source, and other factors affecting living conditions, there is a high degree of population aggregation in these areas.

#### 3.3.4. Location Conditions

**Figure 12:**
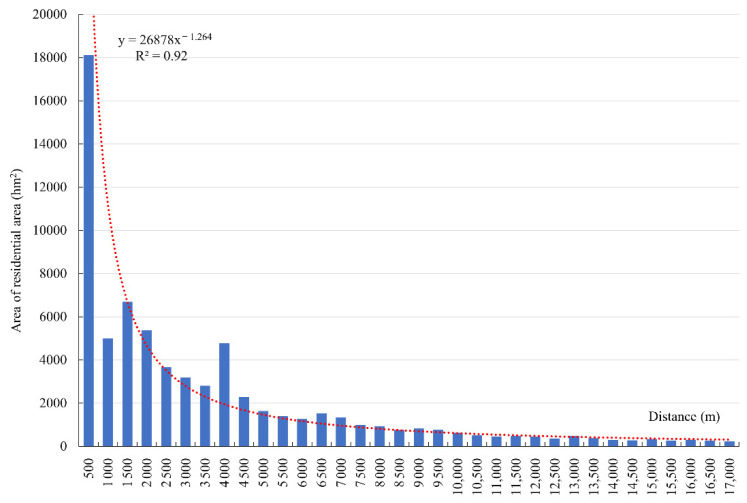
Figure of the total area of residential patch changing with the distance from the road network.

Next, this paper analyzed the buffer zone of the Tibetan traffic network in 500 m intervals. The results indicated that, with an increase in distance from the transportation network, the area distribution of the residential areas in Tibet showed a significant decreasing trend, with some minor fluctuation in some areas (Figure 12). The distribution in different buffer zones showed obvious differences, and 82.27% of residential areas were concentrated within the range of 8 km from a road. Within 500 m, the residential patch area is 23,216.4 ha, accounting for 29.16% of the total patch area. In the range of 500 m to 1000 m, the patch area distribution suddenly decreased to 7627.50 hm^2^, accounting for only 9.58%. It can be found from the figure that in the range of 2500–3000 m, the patch area distribution showed a small fluctuation, accounting for 6.47%, which was slightly higher than that in the ranges of 1000–1500 m and 1500–2000 m. About 14.33% of the residential areas were more than 10 km away from the road. Such residential areas are mostly located in mountainous areas with high altitudes and large slopes, which are mainly affected by the industrial types dominated by animal husbandry, the strong demand in animal husbandry production for good pastures, and the relatively backward road infrastructure conditions. Overall, the distribution of residential areas in Tibet showed significant traffic directivity, mainly due to the increasingly enhanced external communication of local residents and their strong dependence on traffic conditions, most residential areas were distributed within 500 m of a road. With an increase in the distance from the road, the distribution of residential areas became sparse.

## 4. Discussion

### 4.1. Discussion of Water Affinity and Road-Affinity for Tibet Residential Areas

In terms of water affinity, the impact of water on Tibetan residential areas includes two aspects. The first is the material level of water. Water is a powerful resource that benefits people’s life and production, and it is a necessary condition for the existence and development of residents. Therefore, the distribution of residential areas reflects strong hydrophilic characteristics. Second is the spiritual level of water. Some waters are regarded as sacred water by the Tibetan people, including sacred lakes, sacred springs, and rivers endowed with myths and legends. These are places where Tibetans worship and make pilgrimages to. Residential areas adjacent to these waters facilitate folk ritual customs. The spiritual attribute of this water affects the spatial organization of residential areas and various waters, thus forming the unique water landscape of traditional Tibetan residential areas [50].

In terms of road affinity, the spatial distribution of residential areas and traffic have a mutual influence. On one hand, Tibetan residential areas affect the distribution of the road network; on the other hand, the formation of the transportation network has a negative impact on the formation of new residential areas. The interaction between human activities and the environment is a long-term process, and any new type of agricultural and/or economic activity will effectively promote human life at high altitudes [13,49]. The distribution of traditional residential areas in Tibet, to some extent, has affected the distribution of regional traffic routes. After the completion of highway construction, some new residential areas will show strong road affinity characteristics, due to the impact of pastoral re-settlement projects, ecological migration projects, and poverty alleviation projects.

### 4.2. Policy Suggestions on Landscape Planning of Tibetan Residential Areas

The sustainable development of Tibetan settlements is an important part of China’s sustainable socio-economic development, as well as important for frontier security and national defense consolidation [51]. Tibet has a unique natural landscape, cultural foundation, location conditions, and socio-economic conditions, but its fragile ecological environment deserves more attention. The following aspects can be considered when conducting residential landscape planning:

(1) Take the ecological environment as an important constraint condition to promote regional sustainable development. Humans cannot be separated from the natural environment because they depend on it for their survival [52]. The formation and development of residential areas are inseparable from the surrounding ecological environment, where the process of settling residents involves constantly adapting and transforming the surrounding natural environment. The ecological environment in most parts of Tibet is relatively fragile. As such, the unreasonable planning of residential areas will result in an insufficient supply of regional ecosystems, destruction of the ecological environment, and even irreparable damage in some areas. Therefore, the landscape planning of Tibetan residential areas must follow the important principle of ecological security and stability, in order to promote the orderly development of residential areas.

(2) Zoning planning should be carried out according to the landscape’s ecological conditions. According to the specific climate, altitude, hydrology, and soil conditions in Tibet, the ecological function areas can be divided to effectively identify the ecological, production, and living spaces, in order to deal with the contradiction between people and the natural environment in the mixed zone between regions. For example, the “One River and Two Streams” basin (Yarlung Zangbo River, Lhasa River, Nianchu River) and other areas suitable for human habitation should focus on development. Taking advantage of the flat terrain and developed agriculture and animal husbandry, urbanization can be promoted rapidly. For example, Tang et al. [53] used high-resolution satellite images to analyze the impact of rapid urban expansion on sustainable urban development in Lhasa, and proposed a theoretical framework for the “Lhasa development model”. However, it is necessary to carry out ecological relocation and re-settlement for those residential areas in the ecological red line area, with emphasis on highlighting the ecological functions of the area and strictly restricting the random expansion of residential areas.

(3) The effective combination of modern residences and original lifestyles. With the deepening of urbanization, some superior supporting facilities can greatly improve the living conditions of local areas, such as water supply, electricity, heating, networks, and other facilities. Re-planning and re-construction of residential areas can effectively promote the quality of life of local residents; however, the location and scale of the existing residential areas in Tibet are determined by the local people. According to the locally cultivated land resources, grassland resources, and water sources, the space and layout of residential areas have been reasonably arranged, and so, the distribution of such residential areas needs to be preserved to a certain extent. Therefore, when optimizing the layout of residential areas, it is necessary to unify the modern living concept with local conditions and customs, in order to better serve local residents.

## 5. Conclusions 

In this paper, the rank–size principle and landscape indices were used to study the scale and aggregation characteristics of Tibetan residential areas. The superposition analysis method was used to study the factors affecting Tibetan residential areas in four key aspects: altitude, living environment, hydrological conditions, and location conditions. The following conclusions could be drawn:

(1) The Zipf index demonstrated that the distribution of residential patch scale in Lhasa City in central Tibet and in Ngari Prefecture in northwest Tibet was concentrated, with relatively large residential patches being prominent, while the residential patches size of middle and low order having not been developed sufficiently. The residential patch size in these regions is relatively different, while the residential patch size in Hengduan Mountains is relatively balanced.

(2) The landscape index results showed that the counties with an unbalanced distribution of residential area were mainly concentrated in the northwest of Tibet, while the residential area in the counties and districts where the administrative stations of prefecture-level cities (regions) were located presented the polarization phenomenon, with large patches; this polarization phenomenon was most prominent in Lhasa, the capital of Tibet. The distribution of residential areas from southeast to northwest produced a “medium–high–low” pattern. The division degree of residential areas in eastern Tibet was higher than that in northwest Tibet. At the same time, the districts (counties) where the prefecture-level administrative stations are located presented high cohesion characteristics, and those counties with a moderate or high degree of cohesion in residential areas had absolute advantages, in terms of number.

(3) The distribution of Tibetan residential areas was mainly concentrated in the elevation zone of 3500–5000 m, reaching the peak within this range. The residential areas in Tibet presented strong water affinity characteristics, with the total area of residential areas within 500 m of water accounting for one-quarter of the whole of Tibet. This was especially the case in the four river valleys where, due to the good terrain, climate, water sources, and other factors affecting living conditions, the degree of population aggregation is very high. The distribution of residential areas in Tibet is not consistent with that of high-vegetation cover areas, with the NDVI in residential areas mainly ranging between 0.1 and 0.4. With an increase in the distance from the transportation network, the area distribution of Tibetan residential areas showed a significant decreasing trend, with a small increase in the range of 2500–3000 m.

## Figures and Tables

**Table 1:** The selected landscape indices.

Landscape Index	Calculation Formula	Explanation
Degree of landscape division [49] (DIVISION)	$\mathrm{DIVISION}=\left[1-\sum_{i=1}^{n}{\left(\frac{a_{i}}{A}\right)}^{2}\right]$	$a_{i}$ is the area of each residential patch; *A* is the total area of residential patches.
Cohesion index of patch [49] (COHESION)	$\mathrm{COHESION}=\left[1-\frac{\sum_{i=1}^{n}Pi}{\sum_{i=1}^{n}p_{i}*\sqrt{a_{i}}}\right]\cdot \left[1-\frac{1}{\sqrt{Z}}\right](100)$	*P_i_* is the perimeter of the residential patch; *a_i_* is the area of residential patch *i*; *Z* is the total area of the residential area
Patch density (PD)	$\mathrm{PD}=\frac{N}{A}(10,000)$	*N* is the total area of residential patch; *A* is the total area of research area
Largest patch index (LPI) [49]	$\mathrm{LPI}=\frac{max(a_{i})}{A}$	*a_i_* is the area of each residential patch *A* is the total area of residential patch

**Table 2:** Indices related to residential patch size in Tibet.

Region	Zipf Index	R^2^	Ln*S*_0_	Ln*R_i_*
Shannan City	0.833	0.9964	6.9315	1.1961
Xigaze City	0.7323	0.9843	6.6247	1.3441
Nagqu City	0.8551	0.9882	6.4225	1.1556
Nyingchi City	0.8186	0.9843	6.6277	1.2024
Lhasa City	1.0006	0.9927	7.1177	0.9921
Chamdo City	0.6555	0.9917	6.2847	1.5128
Ngari Prefecture	1.4947	0.9813	7.3751	0.6565

In the table above, *S*_0_ is the theoretical value of the first city size; *S_i_* is the residential patch size in the rank order *R_i_*.

**Table 3:** LPI, PSSD, TA Index of residential patches in Tibet.

Region	LPI	PSSD	TA
The whole Tibet area	11.42	317,613.61	795.96
Shannan City	6.97	129,043.14	167.07
Xigaze City	12.86	143,959.17	157.28
Nagqu City	23.4	125,992.14	61.83
Nyingchi City	13.61	135,951.56	81.21
Lhasa City	46.4	738,921.57	195.8
Chamdo City	1.55	31,553.15	102.78
Ngari Prefecture	31.88	162,034.43	29.99

In the table above, LPI is largest patch index; PSSD is standard deviation of patch size; TA is the total area of patch.

**Table 4:** DIVISION and COHESION Index of residential patches in Tibet.

Region	DIVISION	COHESION
Whole Tibet area	0.9849	99.54%
Shannan City	0.9911	99.57%
Xigaze City	0.9820	99.44%
Nagqu City	0.9423	99.33%
Nyingchi City	0.9777	99.51%
Lhasa City	0.7815	99.75%
Chamdo City	0.9982	99.21%
Ngari Prefecture	0.8863	99.63%

In the table above, DIVISION is degree of landscape division; COHESION is cohesion index of patch.

**Table 5:** Area and proportion of residential areas under different NDVI levels.

NDVI	Level	Area of Residential Area	Percentage of Area
0–0.1	I	51.67	6.49%
0.1–0.2	II	185.30	23.28%
0.2–0.3	III	192.97	24.24%
0.3–0.4	IV	121.55	15.27%
0.4–0.5	V	58.70	7.37%
0.5–0.6	VI	133.56	16.78%
0.6–0.7	VII	25.17	3.16%
0.7–0.8	VIII	19.87	2.50%
0.8–0.9	IX	5.23	0.66%
0.9–1	X	1.94	0.24%

## Data Availability

Not applicable.
